# A plate-based high-throughput assay for virus stability and vaccine formulation

**DOI:** 10.1016/j.jviromet.2012.06.014

**Published:** 2012-10

**Authors:** Thomas S. Walter, Jingshan Ren, Tobias J. Tuthill, David J. Rowlands, David I. Stuart, Elizabeth E. Fry

**Affiliations:** a Division of Structural Biology, University of Oxford, The Henry Wellcome Building for Genomic Medicine, Roosevelt Drive, Oxford, OX3 7BN, UK; b Institute for Animal Health, Pirbright Laboratory, Ash Road, Pirbright, Surrey, GU24 0NF, UK; c The Faculty of Biological Sciences, University of Leeds, Leeds, LS2 9JT, UK

**Keywords:** High-throughput, Virus stability, Vaccine formulation

## Abstract

Standard methods for assessing the thermal stability of viruses can be time consuming and rather qualitative yet such data is a necessary requisite for vaccine formulation. In this study a novel plate-based thermal scanning assay for virus particle stability has been developed (PaSTRy: Particle Stability Thermal Release Assay). Two environment-sensitive fluorescent dyes, with non-overlapping emission spectra and different affinities, are used to accrue simultaneously independent data for the overall stability of the virus capsid, as judged by the exposure of the genome, and for capsid protein stability according to the exposure of hydrophobic side chains which are normally buried. This offers a fast and efficient high-throughput method to optimise vaccine formulation and to investigate the processes of virus uncoating.

## Highlights

► A plate-based assay for virus measuring virus stability. ► Two fluorescent dyes measure independently but simultaneously capsid stability and capsid protein stability. ► A fast and efficient high-throughput method to optimise vaccine formulation. ► Facilitates the dissection of virus uncoating.

Determination of the stability of virus particles is critical to the development of vaccines which must remain stable for transport and storage. This issue is especially important in the developing world where longer transport times and the absence of an established cold chain can hamper vaccination programmes. Current methods for assaying particle stability use comparative infectivity with respect to standards to determine the number of infective virions per unit volume (WHO, 2006).

Thermal scanning is a well-established method for the assessment of protein stability, in which the protein sample is slowly heated and its state monitored. Fourier-transform infrared spectroscopy (FTIR (Cooper and Knutson, 1995; Niesen et al., 2008)) and circular dichroism (CD (Benjwal et al., 2006)) have both been used to follow the loss of protein secondary structure as the temperature of the sample is increased, however calorimetry (Brandts and Lin, 1990) and light-scattering (Kurganov, 2002), are more popular due to the ready availability of these instruments.

For soluble proteins the so-called Thermofluor (also known as differential scanning fluorimetry, DSF, or thermal shift assay) (Pantoliano et al., 2000) has proven useful for identifying conditions which change the stability of the protein (Niesen et al., 2008) or for finding more stable homologues and mutants (Lavinder et al., 2009). Thermofluor uses an environment-sensitive fluorescent dye to monitor the exposure of the hydrophobic core of the protein to solvent and hence protein unfolding, and has become popular due to the ease of implementation on conventional quantitative PCR instruments (qPCR) combined with fluorescent dyes originally developed as gel stains, especially SYPRO orange. The use of standard format 96-well plates and relatively small amounts of material make this an attractive method, which has been successfully deployed with proteins for several years by ourselves and others (Ericsson et al., 2006; Geerlof et al., 2006; Sainsbury et al., 2008).

Many viruses comprise an effectively sealed protein shell harbouring the viral genome. It might be expected therefore that the loss of capsid integrity would proceed, perhaps via conformational changes, to the point where the protein shell either undergoes complete dissolution releasing the genome, or reconfigures to such an extent that the genome either escapes or is rendered accessible to dye. In either case, although the protein subunits are unlikely to unfold they may well expose hydrophobic regions concealed in the native capsid. Whilst changes in virus capsid protein association might therefore be monitored using a fluorescent dye such as SYPRO orange, a dye sensitive to the presence of nucleic acid would allow the accessibility of nucleic acid to be directly monitored. The use of two dyes simultaneously would provide a picture of the changes in the protein components, via a series of melting temperatures, *T*_m_s, alongside the temperature at which nucleic acid is released or exposed, *T*_R_, (calculated in the same way as *T*_m_). Such a nucleic acid dye must be sensitive over a broad range of temperature and physical and chemical conditions, have excitation and emission spectra compatible with the instrumentation available, and, critically, be sufficiently large so as not to prematurely penetrate the mature viral capsid through pores or “thermal breathing”, thereby obscuring any change in fluorescence upon capsid opening (Tuthill et al., 2009).

This study sought to devise a thermofluor-type assay to accurately assess the stability of viral capsids. Such an assay could be useful for improving vaccine stability, and as a general tool to examine the dynamics of viral uncoating.

Equine rhinitis A virus (ERAV) was produced and purified as previously described (Tuthill et al., 2009). Briefly, infected Ohio HeLa cells were lysed by freeze–thawing at the peak of virus growth. After centrifugation to remove cell debris virus particles were precipitated with ammonium sulphate and pelleted through a 30% sucrose cushion before separation by ultracentrifugation in a 15–45% sucrose gradient. The virus was located by measuring the absorbance at 260 nm of the fractionated gradient.

The preparation used for the initial proof of principle experiment contained approximately 30% sucrose and contained ERAV at a concentration of approximately 0.04 mg/mL. The investigation of the pH-dependence of particle and capsid protein stability used material from which the sucrose had been removed and which had been concentrated to approximately 0.15 mg/mL in 50 mM HEPES pH 7.3, 50 mM NaCl.

The type 1 Mahoney strain Polio virus (PV) was produced and purified as previously described (Tuthill et al., 2006). Briefly, PV was grown in HeLa cells maintained in suspension culture. The cells were harvested by centrifugation and were subjected to two cycles of freeze–thaw to release the virus before removal of cell debris by low speed centrifugation. The virus was purified by ultracentrifugation in a CsCl density gradient and the fractions containing the virus particles pooled and concentrated.

Bovine enterovirus type 2 (BEV2) was produced and purified as described previously for BEV type 1 (Smyth et al., 1993). Briefly, BHK 21 cell monolayers were infected with BEV2 and the cells and supernatant harvested at maximum cpe. After freeze–thawing the debris was removed by centrifugation and the supernatant made 50% saturated ammonium sulphate to precipitate virus. The resuspended virus was pelleted through a 30% sucrose cushion and finally purified by ultracentrifugation through a 15–45% sucrose gradient.

For the PaSTRy assay, 50 μL volume reactions were set up per well. Typically a small volume of concentrated sample provided 1–2 μg virus per experiment although if the virus concentration was low then a larger volume of sample was required in order to provide a minimum of 1 μg.

Fluorescent dyes were obtained from Molecular Probes (Life Techologies, Paisley, UK); 5000× stocks of both SYPRO orange (product S-6650) and SYPRO red (S-6653), 5 mM stock of SYTO9 (S-34854) and 10,000× of SYBR green II (S-7564), all supplied in dimethylsulphoxide (DMSO). Working stocks of 50× SYPRO orange and SYPRO red, 100× SYBR green II, and 50 μM SYTO9 were produced freshly for each experiment by dilution in milliQ-grade water. The dyes were used at final concentrations of 3× SYPRO orange and SYPRO red, 10× SYBR green II and 5 μM, SYTO9, by diluting the working stocks into the final reaction, or in the case of multiple parallel experiments, by preparation of mastermix solutions containing dyes, buffer and virus sample.

The remainder of the 50 μL reaction volume was made up with buffer, 10 mM HEPES pH 8.0, 200 mM NaCl. By using a large volume of well-buffered screening solution with a small volume of sample it is possible to readily screen a wide range of conditions while diluting the effect of any constituent of the sample buffer.

In order to screen the pH-dependence of ERAV the SPG buffer mixture (Molecular Dimensions, Newmarket, UK) consisting of succinate, phosphate and glycine, (Newman, 2004)) was used at 0.1 M across a pH range of 4.0–9.5 in 0.5 pH unit steps with 0.1 M NaCl in all wells. This buffer mixture contains all ion species at all points in the pH range and so any stabilisation effect of specific ions could be excluded from the analysis.

Typically a dual-dye reaction would contain the following:

**Table:** 

Virus sample (1 mg/mL)	1	*Total 1 *μg
SYPRO red (50×)	3	*3*× *final*
SYTO9 (50 μM)	5	*5* μM *final*
Buffer	41	

Total	50 μL	

For the experiments described below we used two different machines, the Opticon2 (Bio-rad, Hemel Hempstead, UK) and the MX3005p (Agilent, Edinburgh, UK). Reactions were set up in 96-well PCR plates (low profile skirted plates AB-1000/w, Thermo, for the BioRad instrument; semi-skirted plates, product 401334, Agilent, for the Agilent instrument), mixed by pipetting, and then centrifuged at 2000 × *g* to remove any bubbles. The plates were then sealed using optically clear foils (BioRad Microseal B, MSB-1001).

The BioRad Opticon2 qPCR machine uses an array of LEDs for excitation in the 470–505 nm range. Emission is detected on two channels: 523–543 nm and 540–700 nm. Both SYPRO orange and SYTO9 (or SYBR green) produced a stronger signal in the second, broader, channel so this was used for detection of all dyes. This instrument therefore can only be used for experiments with one dye per well. Thermal control is provided by the Peltier unit of a MJ Research DNA Engine which can achieve temperatures below ambient.

The experiments were ramped from 4 to 99 °C, taking a fluorescence reading every 0.5 °C after holding for 10 s, each such run taking approximately one hour to complete. Particular attention was paid to ensure that heating of the sample was not excessive while the lid of the instrument was pre-heated.

The temperatures (*T*_R_, *T*_m_) of the transitions for melting curves were calculated from the inflection point (d*I*/d*T*) of the fluorescence intensity (*I*) as a function of temperature (*T*). When necessary, noisy data were smoothed to aid the identification of the d*I*/d*T* peak and hence assign *T*_R_ or *T*_m_.

The MX3005 qPCR machine uses a quartz tungsten halogen lamp in combination with one of five selectable bandpass filters for excitation; and a single scanning photomultiplier tube with one of five bandpass filters for fluorescence detection. Both the excitation and emission filters, bandwidth ∼10 nm, can be specified as required and are independently selectable in order to allow mismatching. The excitation (ex) and emission (em) bandpass filters used for the dyes were as follows: SYPRO orange, ex: 492 nm, em: 585 nm (∼20% stronger than ex: 492 nm, em: 610 nm); SYPRO red, ex: 585 nm, em: 665 nm (∼30% stronger than ex: 535 nm, em: 665 nm); SYTO9, ex: 492 nm, em: 517 nm. Thermal control for this instrument is also provided by a Peltier unit which in this case can only operate from 25 to 100 °C limiting the low temperature data that can be gathered.

The experiments were ramped from 25 to 99 °C recording in triplicate fluorescence readings for each of the filter combinations specified every 1 °C, taking approximately one hour to complete.

**Fig. 1:**
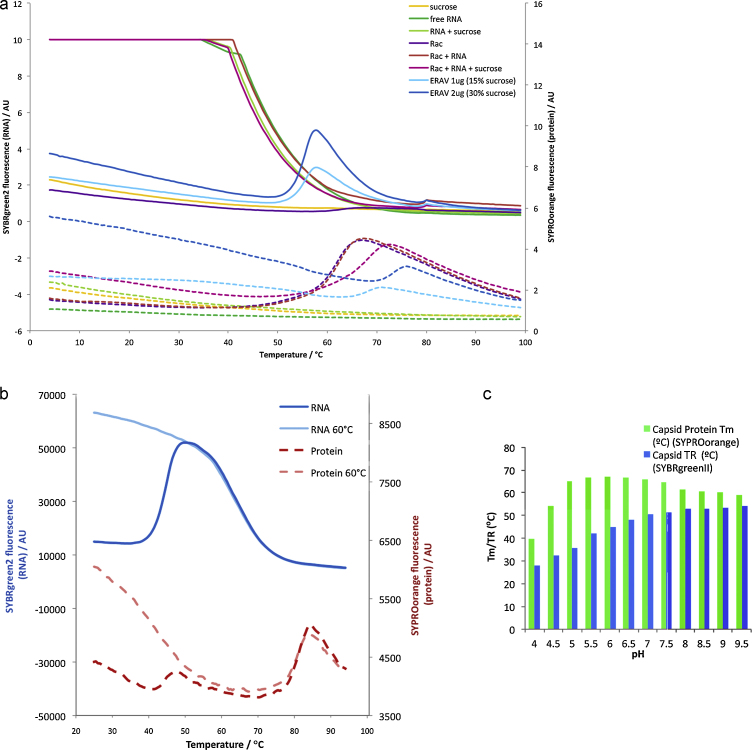
Monitoring protein unfolding (broken-lines) and RNA release (solid lines) using SYPRO orange and SYBR green II (SYTO9 was identical). (a) ERAV (*T*_R_ 55 °C, *T*_m_ 69 °C with 15% sucrose and 74 °C with 30% sucrose). Sucrose stabilises the viral coat proteins but not the capsid. Controls: sucrose, free-RNA and non-viral protein Rac1 (also stabilised by sucrose); (b) poliovirus, *T*_R_ 45 °C, major *T*_m_ 80 °C, note second *T*_m_ coincident with *T*_R_. Control: virus uncoated by pre-heating (10 min, 60 °C). (c) Exposure of ERAV genome and unfolding of capsid proteins vary independently with pH. (a) and (c) used a BioRad Opticon2 and (b) an Agilent MX3005p.

Initial investigations indicated that the RNA-sensitive dyes SYBR green II and SYTO9 were both suitable (data not shown). Proof-of-principle experiments with these dyes used two picornaviruses which show different capsid alterations during the process of cell entry and uncoating; equine rhinitis A virus (ERAV), an aphthovirus often used as a surrogate for foot-and-mouth disease virus, dissociates into pentameric units after release of the RNA (Tuthill et al., 2009), and the enterovirus poliovirus type I, which undergoes a conformational transition to release the RNA genome from an expanded particle (Hogle, 2002). Parallel experiments containing one dye each were set up to monitor both the protein unfolding, via *T*_m_, and the RNA release, via *T*_R_, from the virus capsid (Fig. 1a). For ERAV the temperature of release of nucleic acid, *T*_R_, and the protein melting temperature, *T*_m_, were found to be quite distinct, 55 °C and 69 °C respectively (Fig. 1a) for 1 μg of virus (*T*_m_ rose to 74 °C for 2 μg virus in the presence of greater amounts of sucrose). No *T*_m_ was observed coincident with the release of RNA, indicating that few hydrophobic residues were exposed to solvent upon capsid opening. This finding is in line with the largely polar nature of the interface between pentamers observed in the crystal structure (Tuthill et al., 2009). For poliovirus (Fig. 1b) the picture was broadly similar to ERAV, with a lower *T*_R_ of 45 °C and a major *T*_m_ at 81 °C, however there was another less-pronounced but distinct *T*_m_ at the same temperature as *T*_R_ reflecting the conformational change associated with capsid expansion and exposure of viral RNA. The pH stability of the ERAV capsid and the capsid proteins was also investigated using a broad-range buffer (Newman, 2004). The results (Fig. 1c) confirm that protein unfolding and RNA release data are distinct properties. The stability of the capsid is much reduced in an acidic environment, *T*_R_ falls below body temperature at less than pH 5, consistent with acid-induced uncoating (and inactivation (Tuthill et al., 2009)). In contrast the capsid proteins have peak stability ∼pH 6 and are stable over a broader pH range.

To increase sensitivity and monitor both *T*_R_ and *T*_m_ simultaneously a “dual-dye” protocol was developed. This requires that the emission spectra of the dyes are sufficiently different to avoid cross-talk and also requires a qPCR machine with appropriate band pass filters (e.g. Agilent MX3005p). By substituting the dye SYPRO orange with the similar protein dye SYPRO red the emission spectrum could be monitored near its peak (ex: 585 nm, em: 665 nm). At this wavelength the fluorescence from nucleic acid-bound SYTO9 is undetectable (a small amount of cross-talk is observed for SYBR green II). Similarly by monitoring SYTO9 at 517 nm near its emission peak there is no cross-talk from the fluorescence of SYPRO red (data not shown). Dye concentration is critical to obtaining a good signal, particularly for the protein-sensitive dyes which exhibit collisional quenching at final concentrations greater than ∼4× (data not shown). The RNA-dependent dyes produce a ∼7-fold greater fluorescence signal than the protein-dependent dyes and therefore the release of nucleic acid could potentially be observed using an order of magnitude less virus. Nevertheless, in many cases gathering complementary data simultaneously for both RNA release and protein rearrangement is extremely valuable.

**Fig. 2:**
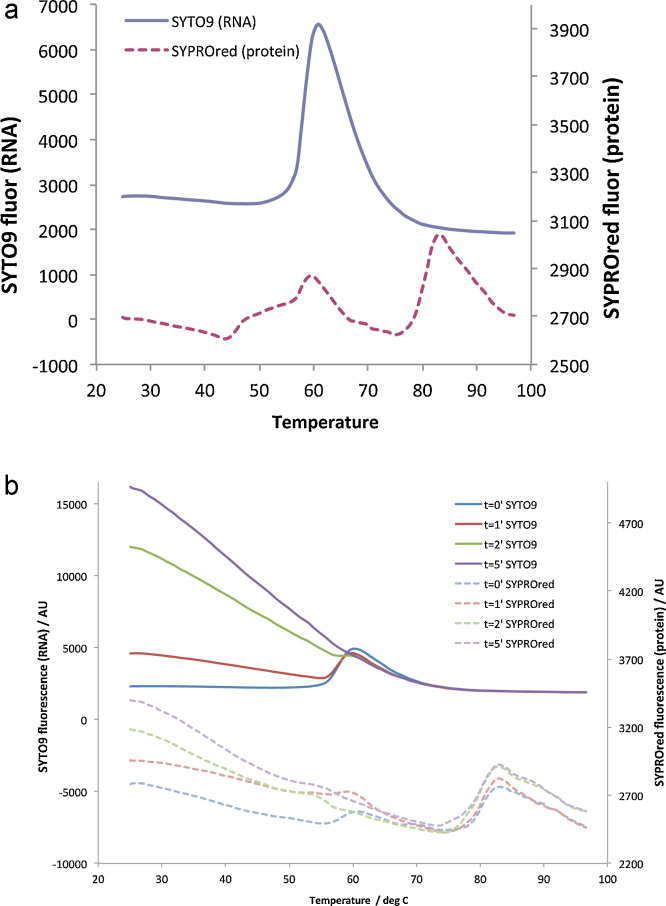
Analysis of mature virus particles of BEV2 with SYPRO red (broken-lines) and SYTO9 (solid lines) performed in dual-dye experiments. (a) Capsid proteins show several *T*_m_ (45, 58, 81 °C), one associated with genome exposure (*T*_R_ 58 °C). (b) Analysis of protocols to capture uncoating intermediates using pre-heating for 0 to 5 min at 56 °C before cooling and performing the assay. With longer incubation a greater proportion of virions uncoated, as indicated by increased initial fluorescence and loss of the *T*_R_ (58 °C) and coincident protein *T*_m_. Both experiments used an Agilent MX3005p.

The effectiveness of this dual-dye system is shown in Fig. 2a, where the stability of particles both containing and lacking RNA for a second enterovirus, BEV2 (Fry, E., Ren, J., Walter, T.S., Rowlands, D., Stuart, D.I., unpublished data) is investigated. In addition to the transition associated with the global unfolding of the capsid proteins (*T*_m_ of ∼80 °C), BEV2 showed, as for poliovirus, a second protein melting transition (*T*_m_ of 58 °C) coincident with the release of RNA (*T*_R_ of 58 °C). BEV2 is clearly more stable than poliovirus (*T*_R_ being elevated by 13 °C). This demonstrates the ability to reliably monitor this transition via the exposure of hydrophobic residues, and is consistent with the previous observations of conformational changes in poliovirus upon heating (Hogle, 2002). Interestingly, for BEV2, there is another transition (*T*_m_ of 46 °C), which remains uncharacterised, before the genome is released. The dual-dye experiment was used to evaluate different pre-heating protocols, providing a quick and straightforward measure of the extent of the modification on the population of virus particles (Fig. 2b).

How robust is the method in the face of the use of different excipients in the formulation? To address this we have performed a full spectral analysis, with scans taken from 0.5 mL samples in a quartz cuvette by a Perkin-Elmer (Waltham, USA) Luminescence Spectrometer LS50B using 3 nm slits for both excitation and emission with a scan speed of 50 nm/min. Yeast RNA extract (8 μg/mL, Sigma–Aldrich, Poole UK) and the detergent dodecyl-beta-maltoside (DDM,1.5 mM, ∼10 × CMC) (Anatrace, High Wycombe, UK) were used to enhance the fluorescence of SYTO9 and SYPROred respectively. All samples contained 0.1 M succinate–phosphate–glycine buffer (SPG, Molecular Dimensions Newmarket, UK; Newman, 2004). We analysed not only our original formulation but a wide range of alternatives. We found that both dyes work across a pH range from 4 to 10, showing no spectral disturbance (although there is marked intensity fall off for SYTO9 at the extremes, data not shown). Thus the method can be used across a broad pH range. We also looked at the effect of high salt (4 M NaCl), sucrose (56%) and PEG3350 (40%) concentrations. In no case was there a significant change in the spectral properties or sufficient loss of efficiency to compromise the method.

**Fig. 3:**
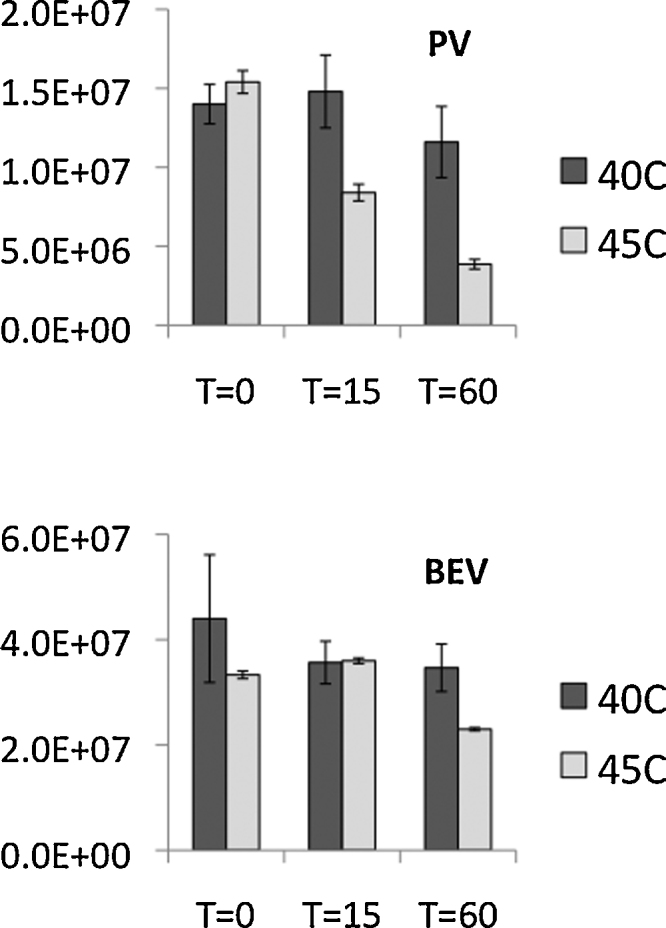
Infectivity of heat-treated particles of BEV2 (labelled BEV) and poliovirus (labelled PV), as estimated by plaque assay after incubation at two temperatures (40 °C and 45 °C) and for times of 0, 15 and 60 min.

Finally we have investigated how the results correlate with conventional infectivity-based assays of thermal stability. We performed experiments with BEV2 and poliovirus. Infected cell culture supernatants were adjusted to approximately equivalent titres by at least ten-fold dilution into cell culture medium (DMEM supplemented with 1% serum, 20 mM HEPES and penicillin and streptomycin at standard concentrations). Samples were incubated at elevated temperatures and times (see Fig. 3) and infectivity titrated by plaque assay. Briefly, confluent monolayers were inoculated with ten-fold serial dilutions of samples and overlaid with medium (as above) containing 0.6% agarose. Monolayers were fixed and stained two days post-infection with 10% ethanol, 3.7% formaldehyde, 1 mg/mL methylene blue. Plaques were counted and titres expressed as pfu/mL. The results are shown in Fig. 3. We would expect *T*_R_ to correspond to the temperature at which the virus becomes inactivated and this temperature is 58 °C and 45 °C for BEV2 and poliovirus respectively. In line with this BEV shows little loss of infectivity at either 40 °C or 45 °C whilst poliovirus shows the expected dramatic loss of infectivity at 45 °C.

In summary, a technique has been devised, termed PaSTRy (protocols available from the authors), using thermal-scanning with fluorescent dyes to characterise virus capsid stability and has been shown to be effective for picornaviruses possessing different mechanisms of genome release. The present protocol allows 96 conditions to be screened in as little as an hour and could be used to fast-track vaccine formulation tests compared to current techniques. In this study it was observed that the method will tolerate a range of excipients in the formulation, but the level of virus purity required has not been investigated systematically. In addition the temperature at which the RNA is made accessible (*T*_R_) appears to be a good surrogate for the temperature at which virus inactivation occurs, as measured by infectivity assay. It may be necessary to use larger or conjugated nucleic-acid sensitive dyes with viruses that have enlarged capsid pores. Nonetheless, this technique should have broad applicability beyond the investigation of the dynamics of virus uncoating and genome release, to the general characterisation of protein-nucleic acid complexes, as well as facilitating sample formulation for the production of more stable vaccines.

## Author contributions

DIS and EEF conceived of the technique. DJR and TJT supplied reagents. TSW designed and performed experiments with DJR, TJT and EEF. All authors discussed the conceptual and practical implications. The manuscript was prepared with input from all authors.

## Competing financial interests

The authors declare that they have no competing financial interests.

## References

[bib0005] Benjwal S., Verma S., Rohm K.H., Gursky O. (2006). Monitoring protein aggregation during thermal unfolding in circular dichroism experiments. Protein Science.

[bib0010] Brandts J.F., Lin L.N. (1990). Study of strong to ultratight protein interactions using differential scanning calorimetry. Biochemistry.

[bib0015] Cooper E.A., Knutson K. (1995). Fourier transform infrared spectroscopy investigations of protein structure. Pharmaceutical Biotechnology.

[bib0020] Ericsson U.B., Hallberg B.M., DeTitta G.T., Dekker N., Nordlund P. (2006). Thermofluor-based high-throughput stability optimization of proteins for structural studies. Analytical Biochemistry.

[bib0025] Geerlof A., Brown J., Coutard B., Egloff M.P., Enguita F.J., Fogg M.J., Gilbert R.J.C., Groves M.R., Haouz A., Nettleship J.E., Nordlund P., Owens R.J., Ruff M., Sainsbury S., Svergun D.I., Wilmanns M. (2006). The impact of protein characterization in structural proteomics. Acta Crystallographica: Section D, Biological Crystallography.

[bib0030] Hogle J.M. (2002). Poliovirus cell entry: common structural themes in viral cell entry pathways. Annual Review of Microbiology.

[bib0035] Kurganov B.I. (2002). Kinetics of protein aggregation. Quantitative estimation of the chaperone-like activity in test-systems based on suppression of protein aggregation. Biochemistry. Biokhimiia.

[bib0040] Lavinder J.J., Hari S.B., Sullivan B.J., Magliery T.J. (2009). High-throughput thermal scanning: a general, rapid dye-binding thermal shift screen for protein engineering. Journal of the American Chemical Society.

[bib0045] Newman J. (2004). Novel buffer systems for macromolecular crystallization. Acta Crystallographica: Section D, Biological Crystallography.

[bib0050] Niesen F.H., Koch A., Lenski U., Harttig U., Roske Y., Heinemann U., Hofmann K.P. (2008). An approach to quality management in structural biology: biophysical selection of proteins for successful crystallization. Journal of Structural Biology.

[bib0055] Pantoliano M., Rhind A., Salemme F. (2000). Microplate thermal shift assay for ligand development and multi-variable protein chemistry optimization. USPTO.

[bib0060] Sainsbury S., Ren J., Saunders N.J., Stuart D.I., Owens R.J. (2008). Crystallization and preliminary X-ray analysis of CrgA, a LysR-type transcriptional regulator from pathogenic *Neisseria meningitidis* MC58. Acta Crystallographica: Section F, Structural Biology and Crystallization Communication.

[bib0065] Smyth M., Fry E., Stuart D., Lyons C., Hoey E., Martin S.J. (1993). Preliminary crystallographic analysis of bovine enterovirus. Journal of Molecular Biology.

[bib0070] Tuthill T.J., Bubeck D., Rowlands D.J., Hogle J.M. (2006). Characterization of early steps in the poliovirus infection process: receptor-decorated liposomes induce conversion of the virus to membrane-anchored entry-intermediate particles. Journal of Virology.

[bib0075] Tuthill T.J., Harlos K., Walter T.S., Knowles N.J., Groppelli E., Rowlands D.J., Stuart D.I., Fry E.E. (2009). Equine rhinitis A virus and its low ph empty particle: clues towards an aphthovirus entry mechanism?. Plos Pathogens.

[bib0080] WHO, 2006. Expert Committee on Biological Standardization: Guidelines on Stability Evaluation of Vaccines. Adopted 2006.

